# Donor Noradrenaline Support Is Not Associated with Decreased Survival in Heart Transplant Recipients

**DOI:** 10.3390/jcm11247271

**Published:** 2022-12-07

**Authors:** Daniel Oehler, Charlotte Böttger, Moritz Benjamin Immohr, Raphael Romano Bruno, Jafer Haschemi, Daniel Scheiber, Fabian Voß, Patrick Horn, Hug Aubin, Igor Tudorache, Ralf Westenfeld, Payam Akhyari, Malte Kelm, Artur Lichtenberg, Udo Boeken

**Affiliations:** 1 Department of Cardiology, Pulmonology and Vascular Medicine, Medical Faculty, Heinrich-Heine University, 40225 Düsseldorf, Germany; 2 Department of Diagnostic and Interventional Radiology, Medical Faculty, Heinrich-Heine University, 40225 Düsseldorf, Germany; 3 Department of Cardiac Surgery, Medical Faculty, Heinrich-Heine University, 40225 Düsseldorf, Germany

**Keywords:** donor noradrenaline support, short-term survival, heart transplantation

## Abstract

Objective: Although the application of higher doses of norepinephrine (NE) in potential organ donors is a frequent reason for heart decline, its associations with outcomes after heart transplantation (HTx) are discussed controversially. Therefore, we aimed to explore donor NE support’s potential impact on outcomes in our single-center heart transplant cohort. Methods: All patients who had undergone HTx in our center between September 2010 and April 2022 (*n* = 241) were screened for eligibility. From those, all patients with complete data on donor NE support (*n* = 238) were included. Recipients were divided into three groups according to their donor NE support: without support (*n* = 26), with low support of 0.01–0.2 µg/kg/min (*n* = 132), and with high support of > 0.2 µg/kg/min (*n* = 80). Receiver operating characteristics (ROC) and Kaplan Meier analysis was used to investigate the association of donor NE support and mortality after heart transplantation. Recipient and donor variables, including peri- and postoperative characteristics, were reviewed and compared. Results: NE support in donors ranged between 0 and 2.94 µg/kg/min (median 0.13 µg/kg/min, IQR 0.05–0.26 µg/kg/min). No association between donor NE support and mortality after HTx was observed (AUC for overall survival 0.494). Neither Kaplan-Meier analysis in survival up to 5 years after transplantation (Log Rank *p* = 0.284) nor group comparisons showed significant differences between the groups. With few exceptions, baseline characteristics in recipients and donors were comparable between the groups. Regarding peri- and postoperative parameters, increasing donor NE support was associated with a longer duration of mechanical ventilation (68 h and 95 h vs. 47 h), longer postoperative IMC/ICU stay (14 vs. 15 vs. 19 days), and a higher need for mechanical life support post-HTx (26% and 39% vs. 12%). Conclusion: In this retrospective analysis, NE support in donors prior to heart transplantation was unrelated to differing survival after heart transplantation. However, higher doses of donor NE were associated with prolonged ventilation, longer duration on IMC/ICU, and a higher need for extracorporeal life support in recipients post-HTx.

## 1. Introduction

Orthotopic heart transplantation is currently the preferred therapy for end-stage heart failure patients. However, prolonged waiting times due to shortages of organ procurements still lead to higher death rates on the waiting list for heart transplantation (HTx). As the quality of the donor’s heart is known to have a tremendous effect on outcome and complications after heart transplantation (HTx) [1], it is crucial to identify parameters associated with survival and outcome after heart transplantation. At least equally important seems to be the de-marginalization of those donors bearing parameters mainly historically associated with the potential risk that would otherwise lead to decline but which are less underpinned by data on outcome after heart transplantation. One of those potential donor risk factors for organ quality with heterogenous data on outcome and survival after heart transplantation is the donor noradrenaline support. Over a long period, donors with high noradrenaline support or support at all were dismissed for acceptance, mainly based on old basic science experiences in dogs showing acute myocardial necrosis when administering high doses (>0.4 µg/kg/min) of noradrenaline experimentally [2], as well as on own clinical experiences, as those recipients depicted worse survival rates. In humans, the neurohumoral disturbances before and closely around the brain death in donors lead to hemodynamic and neuroendocrine alterations, including a catecholamine surge, and in the end, to cardiac damage in donor hearts on a cellular level [3]. After brain death is established, endogenous levels of norepinephrine decrease significantly below basal levels [4], leading to hypoperfusion, which eventually has to be overcome with exogenous norepinephrine administration. However, it is unclear to which extent iatrogenous noradrenaline support could play a role in the cardiac outcome.

Therefore, this study aims to address this by investigating a potential association of donor noradrenaline support, including those with exceptionally high dosage, with morbidity within the index stay and survival up to 5 years after heart transplantation.

## 2. Patients and Methods

### 2.1. Ethics

The study followed the principles of the Good Clinical Practice and the Declaration of Helsinki. All subjects voluntarily participated, and it was approved by the local ethics committee. 

### 2.2. Patients and Study Design

All patients who had undergone HTx in our center between September 2010 and April 2022 (*n* = 241) were screened for eligibility. We then included all patients where information on donor noradrenaline usage and complete data on corresponding peak dose level was given (*n* = 238). Recipients were divided into three groups according to their donor noradrenaline support: Group 1 without noradrenaline support (*n* = 26), group 2 with low peak support of 0.01–0.2 µg/kg/min (*n* = 132), and group 3 with high peak support of >0.2 µg/kg/min (*n* = 80).

### 2.3. Data Collection

Relevant recipient and donor variables were assessed and then compared between the three groups. In addition, recipient and donor characteristics and recipient survival after up to 5 years, including 30 days and one year after transplantation, where applicable, were collected.

### 2.4. Statistical Analysis and Figure Making

Receiver operating characteristics (ROC) and Kaplan Meier analysis was used to investigate the association of donor NE support and survival after heart transplantation. Qualitative (dichotomous) variables have been compared using Pearson’s chi-square test or Fisher’s exact test if its conditions of application were not met. The Yates correction was applied when any of the groups contained a null value for an event. Quantitative variables were compared by a Students’ *t*-test. The tests were performed bilaterally, and the significance threshold was set at 0.05. Statistical analysis was conducted with GraphPad Prism (version 9.4.1, San Diego, CA, USA, www.graphpad.com) and IBM SPSS Statistics Software (SPSS, version 28.0.0.1, IBM Corporation, Armonk, NY, USA). Figures were generated using GraphPad Prism (version 9.4.1, San Diego, CA, USA, www.graphpad.com), Microsoft PowerPoint (Microsoft Corporation, Redmond, Washington, USA) and IBM SPSS Statistics Software (SPSS, version 28.0.0.1, IBM Corporation, Armonk, NY, USA).

## 3. Results

### 3.1. Recipient Data

Baseline characteristics of the recipients were similar between all groups, including parameters of size mismatch and the presence of comorbidities. Furthermore, no statistically significant difference in laboratory values could be detected, including recipients’ creatinine, sodium and potassium levels as well as bilirubin and hemoglobin (see Table 1).

### 3.2. Donor Data

Noradrenaline support in donors ranged between 0 and 2.94 µg/kg/min (median 0.13 µg/kg/min, IQR 0.05–0.26). Donors with no noradrenaline support more often died from hypoxic brain damage (31%), this being significant in comparison to medium noradrenaline levels (23%, *p* = 0.02). Additionally, donors with no noradrenaline support had significantly lower donor sodium levels (144 mmol/L) in comparison to those with medium (149 mmol/L, *p* = 0.04) and high (150 mmol/L *p* = 0.03, resp.) donor noradrenaline support.

All other baseline parameters and laboratory findings were equivalent, including other parameters of size mismatch, left ventricular ejection fraction, donor dobutamine support and comorbidities, donor potassium levels, and lactate dehydrogenase (see Table 2).

### 3.3. Perioperative Morbidity

With respect to peri- and postoperative variables, the groups did not differ in length of postoperative hospital stay, cold or total graft ischemia time, duration of surgery, or need for transfusion of packed red blood cells (see Table 3). However, increasing levels of donor noradrenaline support was associated with a trend toward more prolonged postoperative IMC/ICU stay (14 vs. 15 vs. 19 days, *p* for Group 1 vs. 3 = 0.07) and longer duration of mechanical ventilation after heart transplantation, with significance in comparison of those with no (47 days) to those with medium (68 days, *p* = 0.04) and high support (95 days, *p* = 0.01).

With regard to overall postoperative morbidities, patients did not vary in the occurrence of severe infection or sepsis, the likelihood of kidney failure with hemodialysis post-HTx, the incidence of acute graft rejection (>1R), postoperative neurological complications, or the need for re-thoracotomy post-HTx. However, the need for extracorporeal life support (ECLS) post-HTx was increasing with a higher need for donor noradrenaline support, significant between those with no and those with exceptionally high levels (12 vs. 39%, *p* = 0.01).

### 3.4. Survival

**Figure 1:**
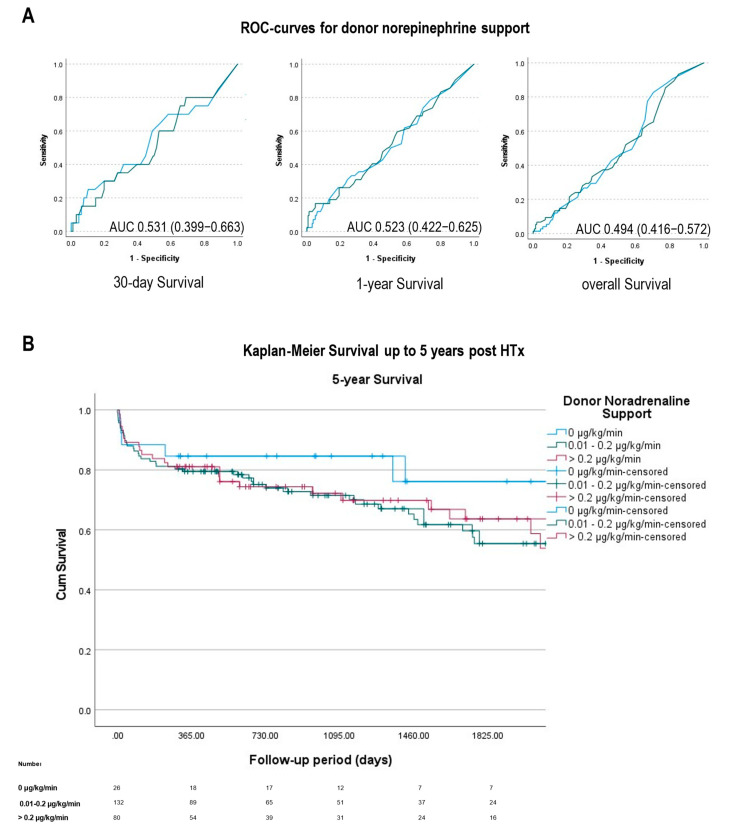
Association of donor norepinephrine support and survival. (**A**) Receiver operating characteristics (ROC) analysis for 30-day, 1-year, and overall survival. The area under the curve (AUC) showed no association for either 30-day (AUC 0.531), 1-year (AUC 0.523), or overall survival (AUC 0.494). (**B**) Kaplan-Meier survivals for no (0 µg/kg/min, *n* = 26), medium (0.01–0.2 µg/kg/min, *n* = 132), or high (>0.2 µg/kg/min, *n* = 80) donor noradrenaline support prior to heart transplantation. Data under the graph representing patients at risk at specific time points in all groups. Log Rank *p* = 0.284.

**Figure 2:**
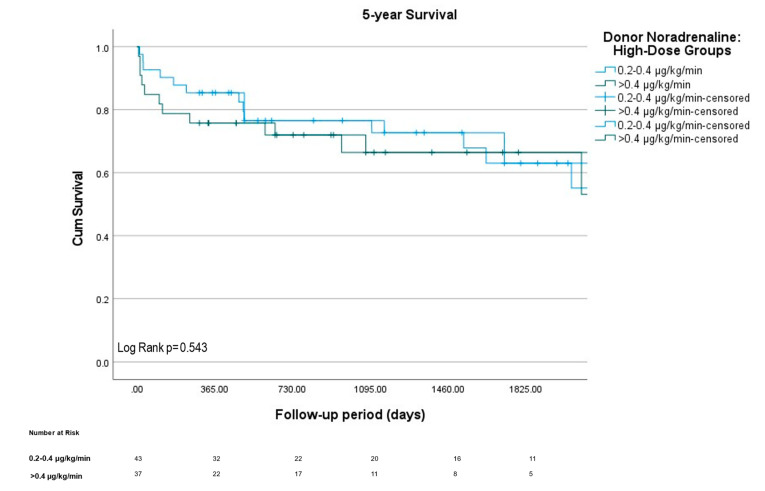
High-dose noradrenaline support subgroup analysis regarding survival. Kaplan-Meier survivals of subgroup analysis between high (0.2–0.4 µg/kg/min, *n* = 43) and exceptionally high (>0.4 µg/kg/min, *n* = 37) donor noradrenaline support prior to heart transplantation. Data under the graph representing patients at risk at specific time points in all groups.

Using receiver operating characteristic (ROC) analysis, no clear general relationship between donor norepinephrine support and survival after HTx was detected (see Figure 1A): AUC for 30-d-Survival 0.531 (95% CI 0.399–0.663), for 1-y-Survival 0.523 (95% CI 0.422–0.625) and overall survival 0.497 (95% CI 0.416–0.572). 30-day and 1-year survival were also comparable between all three groups (Table 1). These findings were supported by a Kaplan-Meier survival analysis (Figure 1B) showing equal distribution of survival across all groups (Log Rank χ^2^ 2.52, *p* = 0.28). Additionally, in a subgroup analysis between high (0.2–0.4 µg/kg/min, *n* = 43) and exceptionally high (>0.4 µg/kg/min, *n* = 37) donor noradrenaline levels, no differences regarding survival in Kaplan-Meier survival analysis could be detected either (Figure 2, Log Rank χ^2^ 0.37, *p* = 0.543).

## 4. Discussion

Limited information is available on the association of perioperative morbidity and survival in heart transplant recipients and the level of donor noradrenaline support prior to heart transplantation. We, therefore, aimed to enlighten possible differences between levels of donor noradrenaline support on outcome after heart transplantation, and thus retrospectively analyzed 238 HTx recipients in a 10-year study period.

Here, we report two main findings: First, despite differing donor baseline characteristics, donor noradrenaline levels had no effect on short-term survival up to one year after heart transplantation. Second, however not influencing survival, increasing noradrenaline levels in donors were associated with worse short-term postoperative outcomes after heart transplantation.

In the current study, we could not observe an apparent association between survival up to 5 years and donor noradrenaline levels. Compared to the literature, 30-day survival in our cohort (ranging from 89–92%) was comparable to larger HTx-cohorts (89–93% [5,6]), and equal 30-day and 1-year survival distribution between all three groups could be seen. The influence of donor noradrenaline usage and their respective levels on recipient survival after heart transplantation remains controversial in the published literature, and only a few studies explicitly analyze this parameter [7,8,9]. While Murana et al. suggest an impact on recipient in-hospital mortality survival [8], this could not be validated in both data from cohort studies by Benck et al. [10] as well as Kutschmann et al. [11]. Interestingly, in a larger study by Angleitner et al. [12], no effect on early survival of recipients could be observed, even in exceptionally high donor noradrenaline levels (>0.4 µg/kg/min). This is in line with our subgroup analysis between those with high (0.2–0.4 µg/kg/min) and exceptionally high levels (>0.4 µg/kg/min), also showing no difference in survival. However, all those reference cohorts included retrospective patient data from different heart transplantation eras, leading to a potential bias. Data for other solid organ transplantations, such as kidney, are also limited but show no general effect of noradrenaline on delayed graft function [13]. Interestingly, in one retrospective liver transplant cohort from Spain, Cuende et al. could show a potential benefit of low-dosage donor noradrenaline support on graft survival [14], and the authors explain that by balancing the hypoperfusion-induced organ damage after brain death.

The usage of donors with noradrenaline support differs regionally, and the completion of data is limited. The majority of all donors in our study had noradrenaline support (89.1%, *n* = 212), which is in line with published data from a cohort in Brazil (89.4%) [15]. However, often only information on donors with <0.1 µg/kg/min is available, while the percentage of those without any support is missing. Zuckermann et al. reported in a large cohort dataset from the Eurotransplant area that around 58% of all recipients had donors with ≥0.1 µg/kg/min noradrenaline support prior to transplantation [9], while others reported rates of approximately 53–60% [7,8]. In our study cohort, 55% of all patients (*n* = 131) had support ≥0.1 µg/kg/min, which was also in the reported range of previous studies. It is worthy of note that in our cohort, the percentage of patients with support ≥0.2 µg/kg/min (34%, *n* = 80) was higher than in an older multicentre study from Kutschmann et al. (approximately 13%) [11]. A more liberal acceptance of donors can partly explain this with higher donor noradrenaline support currently, leading to a potential bias.

Additionally, D’Aragon et al. recently showed (using data of the Canadian DONATE study [16]) that donors with a neurological determination of death (donation after brain death, DBD) had more frequent noradrenaline usage before solid organ donation than those with a circulatory determination of death (DCD, 67 vs. 32%). Due to legal reasons, only donors with a neurological determination of death can be accepted for transplantation in our center; thus, we cannot exclude a potential bias in our analysis. In the whole Eurotransplant area, the absolute number of donors with DCD used for heart transplantations was low (*n* = 4, Annual Report 2019), and in the mentioned study, no heart transplantation at all was performed from donors with a circulatory determination of death. Thus, the potential confounder bias of DCD vs. DBD on outcome after HTx in our reference cohorts seems to be negligible.

Regarding baseline data, the recipients’ preoperative parameters were similar between the groups. Regarding the donors, in the present study, donors with no noradrenaline support more often died from hypoxic brain damage. Recently, it could be shown that intracerebral bleeding (ICB) but not hypoxic brain damage or (ischemic) cerebrovascular events such as donor cause of death were associated with reduced recipient’ survival after heart transplantation [17]. In our cohort, ICB as donor cause of death was not statistically different between donor noradrenaline groups. Additionally, the donor noradrenaline levels in the mentioned study on donor cause of death were comparable between all groups, altogether suggesting no relevance of the reported differences in our study cohort, and partly explainable by size limitations by the low absolute numbers in the group with no donor noradrenaline support (*n* = 26). In addition to the previous difference, in our cohort, only donors with noradrenaline support had mildly elevated sodium levels (median 149 mmol/L and. 150 mmol/L), while those without had normonatremia (median 144 mmol/L). In general, cut-off values for hypernatremia severity range between >150 mmol/ [18] and >156 mmol/L [19]. There is controversial and limited data on the impact of donor sodium dysregulation on patient survival after solid organ transplantation, with cut-off values ranging from 159 to 170 mmol/L [20,21]. Therefore, in our cohort, those with high donor norepinephrine support have elevated but most likely not relevant mild hypernatremia without relevant influence on outcome or survival; however, this is limited obviously through the stated lack of data available on this topic. Currently, there is no data concerning the direct association of hypernatremia and noradrenaline levels; however, we cannot exclude a potential confounder effect here.

Concerning perioperative morbidity, increasing donor noradrenaline levels were associated with a trend towards longer postoperative IMC/ICU stay in our cohort. This is in line with published data from Angleitner et al. [12], where they could also observe a trend towards higher rates of prolonged ICU stay in those with higher donor noradrenaline support compared to those without (28.5% vs. 18.9%). Additionally, high donor noradrenaline support in our cohort was associated with a significantly longer duration of mechanical ventilation (95 h vs. 47 h in those with no support). Although prolonged ventilation is generally defined as longer than 7 postoperative days (168 h) after HTx [12], the absolute difference between the groups seems to be clinically meaningful. Interestingly, the need for extracorporeal life support (ECLS) post-HTx was increasing with a higher need for donor noradrenaline support, being significant between those with no and those with exceptionally high levels (12 vs. 39%, *p* = 0.01). This could be seen as a possible explanation of “coping” the worse effect of higher noradrenaline levels on recipients outcome. ECLS hereby could act as a “life saver”, balancing survival in recipients, as today ECLS therapy is more common than in past transplant eras. This observation also leads to a potential socioeconomic aspect as, although survival is not significantly reduced, the potential in-hospital costs are likely to increase dramatically through a need for ECLS therapy by accepting donors with higher noradrenaline levels.

Moreover, we could see in those patients a trend towards a higher need for blood transfusions, more acute allograft rejection, and a higher percentage of re-thoracotomy post-HTx. These findings suggests a higher short-term risk of cardiocirculatory failure and can be seen as an indicator of a worse disease state after heart transplantation in those recipients with higher donor noradrenaline levels.

From a pathophysiological perspective, several potential pathways could link the amount of donor noradrenaline support to the outcome of the recipient. Older data from animal experiments in dogs showed acute myocardial necrosis when administering high noradrenaline doses (>0.4 µg/kg/min)^2^. The neurohumoral disturbances before and closely around brain death in human donors lead to hemodynamic and neuroendocrine alterations, including a catecholamine surge, and in the end to cardiac damage on a cellular level ^3^. Endogenous levels of norepinephrine decrease significantly below basal levels ^4^ after brain death, leading to hypoperfusion and the potential need for exogenous norepinephrine administration. However, it is uncertain to what degree the additional amount of iatrogenous dosage on top of the endogenous catecholamines could affect the cardiac outcome. Also, through the administration of vasoconstrictors, the distribution of the cardioplegic solution could be potentially hindered, especially for the blood flow within the coronary arteries; however, currently no data is available to undermine this hypothesis. Additionally, one cannot exclude that noradrenaline levels are a secondary indicator of a more severe disease state of the donor, thereby causing secondary damage to the donor’s heart before heart transplantation. Therefore, most likely, the influence of donor noradrenaline levels on recipient outcomes is a combined effect of both primary and secondary damages to the donor’s heart. Some or all of those potential mechanisms could be responsible for the reduced outcome we see in recipients with high donor noradrenaline; however, we cannot technically prove this hypothesis due to the nature of this study.

The major and obvious limitation of the current study is that it is single-center and includes only retrospective data. Therefore, with larger cohorts, preferably from the most recent era of heart transplantation and possibly with a prospective design, future studies will need to confirm or reject the association of donor norepinephrine support with outcome and survival after heart transplantation.

## 5. Conclusions

In this retrospective analysis, norepinephrine support in donors prior to heart transplantation was unrelated to differing survival after heart transplantation. However, higher doses of donor norepinephrine were associated with prolonged ventilation, longer duration on IMC/ICU, and a higher need for extracorporeal life support in recipients post-HTx.

## Figures and Tables

**Table 1:** Preoperative recipient parameters. Preoperative recipient data regarding the donor noradrenaline support: No (0 µg/kg/min, *n* = 26), medium (0.01–0.2 µg/kg/min, *n* = 132) or high (>0.2 µg/kg/min, *n* = 80). Shown are median values with interquartile range (Q1–Q3) for continuous variables or percentages for discrete variables. Significance was calculated using the Pearson chi-square test or, if its conditions of application were not met, the Fisher’s exact test for qualitative variables and the *t*-test for quantitative variables.

Recipient Variables	All Patients	Gr 1 (0 μg/kg/min)	Gr 2 (0.01–0.2 µg/kg/min)	Gr 3 (>0.2 µg/kg/min)	*p*		
	*n* = 238	Total *n* = 26	Total *n* = 132	Total *n* = 80	1 vs. 2	1 vs. 3	2 vs. 3
Age (y)	58 (50–62)	60 (42–63)	58 (50–63)	59 (53–62)	0.59	0.40	0.54
Gender (% male)	72.2	76.9	75.0	67.5	>0.99	0.46	0.27
Height (cm)	175 (170–180)	177 (172–180)	174 (170–180)	175 (168–180)	0.58	0.33	0.48
Weight (kg)	78 (68–87)	78 (70–88)	78 (67–88)	78 (68–87)	0.91	0.92	0.99
Body mass index (kg/m^2^)	25 (23–28))	24 (22–29)	25 (23–28)	26 (23–29)	0.96	0.79	0.73
Predicted heart mass ratio (%)	1.0 (0.9–1.1)	0.9 (0.8–1.1)	0.97 (0.87–1.08)	0.99 (0.91–1.11)	0.24	0.06	0.17
Cardiac reoperation (%)	63.1	57.7	61.4	67.5	0.83	0.48	0.38
High-urgency waiting list (%)	44.8	50.0	46.2	41.3	0.83	0.50	0.57
Ventricular assist device (%)	50.6	42.3	52.3	51.3	0.40	0.50	0.89
CPR pre-HTx (%)	12.9	15.4	10.6	15.2	0.82	>0.99	0.38
Diabetes mellitus (%)	22.6	30.8	19.8	25.3	0.30	0.61	0.39
Arterial Hypertension (%)	57.1	61.5	54.5	60.8	0.53	>0.99	0.39
ICM (%)	43.2	46.2	38.6	47.5	0.52	>0.99	0.20
Laboratory values							
Hemoglobin (g/dL)	12.0 (10.1–13.6)	12.3 (10.3–13.4)	12 (10.1–13.6)	11.8 (9.8–13.7)	0.95	0.94	0.84
Creatinine (mg/dL)	1.2 (1–1.6)	1.3 (1.1–1.7)	1.2 (1–1.6)	1.2 (1–1.5)	0.33	0.67	0.56
GFR pre-HTx (mL/min)	62 (45–82)	57 (41–77)	64 (46–85)	62 (47–79)	0.47	0.46	0.93
Bilirubin (mg/dL)	0.6 (0.4–1.0)	0.7 (0.4–1.5)	0.6 (0.4–1)	0.6 (0.4–0.9)	0.56	0.25	0.37
Lactate dehydrogenase (U/L)	254 (213–314)	228 (196–306)	253 (215–292)	265 (218–365)	0.47	0.12	0.26
Sodium (mmol/L)	138 (136–141)	138 (136–141)	138 (136–141)	138 (136–140)	0.53	0.85	0.31
Potassium (mmol/L)	4.3 (3.9–4.6)	4.2 (3.8–4.4)	4.3 (3.93–4.6)	4.2 (3.9–4.6)	0.06	0.24	0.34

Abbreviations: CPR = Cardiopulmonary resuscitation; ICM = Ischemic Cardiomyopathy; GFR = Glomerular Filtration Rate.

**Table 2:** Preoperative donor parameters. Preoperative donor data regarding the donor noradrenaline support: No (0 µg/kg/min, *n* = 26), medium (0.01–0.2 µg/kg/min, *n* = 132) or high (>0.2 µg/kg/min, *n* = 80). Shown are median values with interquartile range (Q1–Q3) for continuous variables or percentages for discrete variables. Significance was calculated using the Pearson chi-square test or, if its conditions of application were not met, the Fisher exact test for qualitative variables and the *t*-test for quantitative variables. Bold *p*-values stand for statistical significance.

Donor Variables	All Patients	Gr 1 (0 μg/kg/min)	Gr 2 (0.01–0.2 µg/kg/min)	Gr 3 (>0.2 µg/kg/min)	*p*		
	*n* = 238	Total *n* = 26	Total *n* = 132	Total *n* = 80	1 vs. 2	1 vs. 3	2 vs. 3
Age (y)	46 (35–53)	39 (31–49)	46 (36–54)	46 (33–52)	0.06	0.14	0.56
Gender (% male)	55.0	53.8	56.1	53.8	>0.99	>0.99	0.78
Height (cm)	175 (168–180)	175 (168–184)	175 (169–180)	174 (168–180)	0.67	0.69	>0.99
Weight (kg)	80 (70–85)	79 (65–84)	80 (70–85)	80 (70–89)	0.17	0.06	0.40
Left ventricular ejection fraction (%)	60 (55–65)	61 (55–63)	60 (57–65)	60 (52–65)	0.19	0.81	0.11
CPR pre-brain death (%)	28.2	26.9	28.0	28.8	>0.99	>0.99	>0.99
Norepinephrine, peak dose (µg/kg/min)	0.12 (0.05–0.26)	0 (0–0)	0.1 (0.04–0.15)	0.36 (0.26–0.55)	**<0.0001**	**<0.0001**	**<0.0001**
Dobutamin, peak dose (µg/kg/min) *	4.0 (3.0–4.0)	4.0 (3.0–4.0)	4.0 (2.8–4.0)	4.0 (3.6–4.0)	0.50	0.82	0.28
Arterial Hypertension (%)	50.4	42.9	50.7	52.6	0.77	0.76	>0.99
Donor cause of death							
ICB (%)	46.2	38.5	50.7	52.5	0.67	0.26	0.26
Trauma (%)	22.7	26.9	43.9	21.3	0.80	0.59	0.87
Hypoxic (%)	16.0	30.8	22.7	17.5	**0.02**	0.17	0.31
Vascular (%)	6.3	3.8	12.1	2.5	0.48	>0.99	0.09
Other (%)	8.8	0.0	9.1	6.3	0.08	0.33	0.24
Laboratory values							
Hemoglobin (g/dL)	9.9 (8.2–12)	10.3 (9–12.7)	9.8 (8.2–11.8)	9.8 (8.2–11.8)	0.43	0.31	0.69
Sodium (mmol/L)	149 (144–154)	144 (141–152)	149 (144–154)	150 (144–155)	**0.04**	**0.03**	0.80
Potassium (mmol/L)	4.1 (3.8–4.5)	4.2 (3.8–4.5)	4 (3.7–4.4)	4.2 (3.9–4.6)	0.54	0.63	0.12

Abbreviations: CPR = Cardiopulmonary resuscitation; ICB = Intracerebral Bleeding. * Information on donor dobutamine support available for 43 of 238 patients (data completeness Gr 1: 5/26, Gr 2 27/132, and Gr 3 11/80).

**Table 3:** Peri- and postoperative parameters and survival, grouped by the donor noradrenaline support prior to heart transplantation: No (0 µg/kg/min, *n* = 26), medium (0.01–0.2 µg/kg/min, *n* = 132) or high (>0.2 µg/kg/min, *n* = 80). Shown are median values with interquartile range (Q1–Q3) for continuous variables or percentages for discrete variables. Significance was calculated using the Pearson chi-square test or, if its conditions of application were not met, the Fisher exact test for qualitative variables and the *t*-test for quantitative variables. Bold *p*-values stand for statistical significance.

Outcome and Survival	All Patients	Gr 1 (0 μg/kg/min)	Gr 2 (0.01–0.2 µg/kg/min)	Gr 3 (>0.2 µg/kg/min)	*p*		
	*n* = 238	Total *n* = 26	Total *n* = 132	Total *n* = 80	1 vs. 2	1 vs. 3	2 vs. 3
Total graft ischemia time (min)	213 (186–237)	207 (172–235)	218 (192–242)	206 (186–236)	0.22	0.55	0.52
Graft cold ischemia time (min)	148 (126–172)	145 (119–168)	154 (133–178)	143 (126–167)	0.18	0.51	0.45
Postoperative hospital stay (d)	36 (26–51)	31 (24–40)	34 (26–53)	40 (29–54)	0.18	0.13	0.74
Postoperative IMC/ICU stay (d)	16 (10–28)	14 (11–21)	15 (9–28)	19 (10–30)	0.10	0.07	0.72
Mechanical ventilation (h)	67 (25–176)	47 (14–87)	68 (25–169)	95 (33–197)	**0.04**	**0.01**	0.24
Duration of surgery (min)	413 (341–506)	388 (343–493)	411 (331–513)	427 (363–490)	0.80	0.38	0.34
Blood transfusions (during surgery)							
Packed red blood cells, mL	2970 (1620–4590)	2970 (1080–4050)	2700 (1620–4523)	3240 (1688–5063)	0.56	0.43	0.70
Platelets, mL	880 (660–1540)	880 (440–1485)	880 (660–1540)	880 (495–1540)	0.14	0.25	0.78
Fresh frozen plasma, mL	1000 (0–2000)	1250 (250–1750)	1000 (63–2000)	1250 (0–2438)	0.57	0.22	0.37
Blood transfusions (on IMC/ICU)							
Packed red blood cells, mL	1890 (810–4320)	1350 (743–3038)	1890 (810–4320)	2025 (1013–4725)	0.32	0.15	0.54
Platelets, mL	220 (0–1100)	0 (0–275)	220 (0–990)	440 (0–1375)	0.86	0.53	0.28
Fresh frozen plasma, mL	3500 (2000–7000)	2500 (1500–5250)	3750 (2000–6750)	4000 (2000–8188)	0.30	0.22	0.80
Postoperative morbidity							
Infection/Sepsis (%)	22.6	23.1	23.8	20.5	>0.99	>0.99	0.61
Rejection within stay (%)	6.4	3.8	6.2	7.7	>0.99	0.68	0.78
Hemodialysis post-HTx (%)	57.2	44.0	58.2	60.0	0.27	0.24	0.88
Neurological complications (%)	15.0	11.5	16.2	14.1	0.77	>0.99	0.84
Re-thoracotomy post-HTx (%)	29.9	23.1	28.5	34.6	0.64	0.34	0.44
ECLS post-HTx (%)	28.6	11.5	26.2	38.5	0.13	**0.01**	0.09
Survival							
30-day survival *n* (%)	216/238 (90.8)	23/26 (88.5)	119/130 (91.5)	71/79 (89.9)	0.71	>0.99	0.81
1-year survival *n* (%)	161/203 (79.3)	18/22 (81.8)	89/113 (78.8)	54/68 (79.4)	0.79	>0.99	>0.99

Abbreviations: IMC/ICU = Intermediate Care Unit / Intensive Care Unit; ECLS = Extracorporeal Life Support.

## Data Availability

The data presented in this study are available on request from the corresponding author. The data are not publicly available due to ethical reasons.

## Abbreviations

HTx	Heart Transplantation
vs.	versus
ECLS	Extracorporeal life support
CPR	Cardiopulmonary resuscitation
IMC	Intermediate Care Unit
ICU	Intensive Care Unit
